# Combining enhanced spectral resolution of EMG and a deep learning approach for knee pathology diagnosis

**DOI:** 10.1371/journal.pone.0302707

**Published:** 2024-05-07

**Authors:** Ateka Khader, Ala’a Zyout, Amjed Al Fahoum

**Affiliations:** Biomedical Systems and Informatics Engineering Department, Yarmouk University, Irbid, Jordan; Chongqing University Three Gorges Hospital, CHINA

## Abstract

Knee osteoarthritis (OA) is a prevalent, debilitating joint condition primarily affecting the elderly. This investigation aims to develop an electromyography (EMG)-based method for diagnosing knee pathologies. EMG signals of the muscles surrounding the knee joint were examined and recorded. The principal components of the proposed method were preprocessing, high-order spectral analysis (HOSA), and diagnosis/recognition through deep learning. EMG signals from individuals with normal and OA knees while walking were extracted from a publicly available database. This examination focused on the quadriceps femoris, the medial gastrocnemius, the rectus femoris, the semitendinosus, and the vastus medialis. Filtration and rectification were utilized beforehand to eradicate noise and smooth EMG signals. Signals’ higher-order spectra were analyzed with HOSA to obtain information about nonlinear interactions and phase coupling. Initially, the bicoherence representation of EMG signals was devised. The resulting images were fed into a deep-learning system for identification and analysis. A deep learning algorithm using adapted ResNet101 CNN model examined the images to determine whether the EMG signals were conventional or indicative of knee osteoarthritis. The validated test results demonstrated high accuracy and robust metrics, indicating that the proposed method is effective. The medial gastrocnemius (MG) muscle was able to distinguish Knee osteoarthritis (KOA) patients from normal with 96.3±1.7% accuracy and 0.994±0.008 AUC. MG has the highest prediction accuracy of KOA and can be used as the muscle of interest in future analysis. Despite the proposed method’s superiority, some limitations still require special consideration and will be addressed in future research.

## Introduction

Knee osteoarthritis (OA) is a joint disorder that causes pain and disability, particularly in the elderly [1]. OA results from wear, tear, and deterioration of the articular cartilage [2]. However, OA affects not only the cartilage but is a disease that affects all of the tissue of the joint [3]. There are many reasons for this joint disease, including aging, obesity, injuries, stress, and other factors that weaken the muscles around the joint [4]. The symptom that usually leads to discovering OA is severe pain in the knee.

The knee joint is complex and comprises bone, ligaments, tendon muscles, cartilage, and fluid. The knee joint plays a role in the movement and stability of the human body, shock absorption, and supporting the body weight [5]. Many methods have been used to detect knee abnormalities, including X-ray, magnetic resonance imaging (MRI), and Computer Tomography (CT) [6–9]. In the initial investigation of knee pain, X-ray imaging often serves as the primary diagnostic tool due to its accessibility and cost-effectiveness. However, while X-rays can reveal changes in bone structure and joint space narrowing indicative of osteoarthritis, their capacity to detail the condition of soft tissues, such as cartilage, is limited [10]. This limitation is significant given that knee osteoarthritis (KOA) is characterized not only by bone deterioration but also by the degeneration of cartilage and other joint tissues. Advanced imaging modalities like magnetic resonance imaging (MRI) and computer tomography (CT) scans offer comprehensive insights into both bone and soft tissue states but come with higher costs and increased examination time, potentially limiting their routine use in clinical practice [10]. Furthermore, Inertial Measurement Units (IMUs), including gyroscopes and accelerometers, have been explored for their potential in diagnosing knee pathologies through the analysis of movement patterns [11–13].

Electromyography (EMG) is a valuable, economical and non-invasive technique that complements existing diagnostic tools by providing insights into muscle activity patterns affected by the disease [14]. EMG signals are widely used in different fields, including rehabilitation medicine, human-machine interface design, prosthesis and robotics control, and clinical diagnosis [15–17]. In assessing knee joint function and pathology, various imaging techniques, including fluoroscopy, offer valuable insights, particularly in dynamic conditions [18, 19]. While these methods provide detailed anatomical information during motion, they primarily focus on structural aspects of the knee joint. EMG, on the other hand, complements these imaging techniques by offering unique insights into muscle activity patterns, which are crucial for understanding the functional implications of knee pathologies such as OA [20, 21]. The strength of EMG lies in its ability to capture the neuromuscular dynamics associated with knee movement, providing a direct link to the muscular adaptations or impairments resulting from or contributing to KOA. This capability makes EMG a critical tool in a multidisciplinary approach to diagnosing and understanding KOA, alongside conventional imaging methods. However, the EMG signal is a complex signal affected by intrinsic and extrinsic noise due to motion artifacts, baseline noise, and interference noise [22]. This demands sophisticated processing analysis techniques to extract a significant insight into muscle-specific changes associated with KOA. Despite it’s complexity, the usefulness of EMG technology remains to be invaluable in understanding the muscle state and function. So using EMG signals for classifying the pathological knee by traditional methods could be challenging. Developing an automated system could help diagnose the pathological knee from the EMG data.

Machine learning and deep learning have been used in many biomedical applications [23–26]. They were also utilized to predict and evaluate OA using MRI [27, 28] and X-ray [29, 30]. Furthermore, EMG signals have been investigated using machine learning techniques [31, 32]. Vijayvargiya et al. [33] used EMG for the knee muscles during gait, standing, and sitting positions for normal and abnormal subjects to predict the abnormality. Different machine learning classifiers are used, including the k-nearest neighbor, support vector machine, decision tree, random forest, and extra tree, with the different tree classifiers having the highest accuracy (91%) [33]. Another study used the same data to detect knee abnormalities [32]. Anomaly detection methods have removed abnormal data with the light gradient boosting machine to reach 98% accuracy.

Furthermore, it has been shown that EMG signals can predict lower limb activities for normal and OA patients using a hybrid deep learning model [34]. Interestingly [32–34], studies have used the same data sets, which comprise EMG signals for 11 normal subjects and 11 subjects with knee OA. The data have been taken from the biceps femoris (BF), vastus medialis (VM), rectus femoris (RF), and semitendinosus (ST) muscles while the subject is walking, sitting, and standing. Moreover, in all these studies, the EMG signals from individual muscles were not examined separately. Instead, features were extracted from all four muscles, then the selected features were fed into different machine-learning classifiers. These studies often focused on isolated muscle analysis or conventional machine learning algorithms, which may not fully capture the intricate patterns of neuromuscular activity associated with KOA. Our study extends this body of work by employing a novel integration of high-order spectral analysis (HOSA) with deep learning (specifically, the ResNet101 architecture), aimed at enhancing the diagnostic precision of EMG signal analysis for KOA. Unlike previous efforts, our approach considers the complex, nonlinear interactions between muscle activities, leveraging the advanced feature extraction capabilities of HOSA and the sophisticated pattern recognition power of deep learning.

HOSA method, such as bispectrum and bicoherence, provides higher resolution than low-order power spectral analysis approaches [35]. These methods can capture nonlinear and non-Gaussian properties to comprehend biological signals’ frequency content and interactions. Nonlinear characteristics such as phase coupling, amplitude modulation, and frequency modulation are present in various biological signals [36]. These nonlinearities are crucial to understanding the underlying physiological processes and disorders, and they can be detected and quantified using the HOSA technique [35]. Conventional spectrum analysis techniques can occasionally obscure or overlook complex information in biomedical signals. In order to better analyze and classify data, algorithms for HOSA can extract more features and reveal signals’ latent correlations [37]. Methods based on HOSA analysis have shown promise for improving the categorization and recognition of biomedical signals [23, 25, 35–37].

This study aims to apply deep learning and incorporate bicoherence for the first time to EMG data using HOSA to diagnose KOA as a source of significant ramifications. It can facilitate a more precise diagnosis, allowing knee pathology to be diagnosed and treated sooner. Early diagnosis is the key to effectively controlling OA and halting further joint decline. In addition, it can shed light on how OA develops and the causes of the neuromuscular alterations that characterize the condition. The proposed method will develop more effective treatments and recovery protocols with this knowledge. Moreover, by utilizing EMG data, the proposed method may further reduce the cost, hazards due to imaging, and surgical intervention of knee pathology diagnosis. The contribution of this work can be validated through the comparison of the performance of the proposed method to that of other credible EMG analysis methods and clinical evaluations. The decision to study the EMG signal from the BF, MG, VM, RF, and ST stems from the objective to capture a comprehensive view of the knee’s neuromuscular function. By analyzing signals from these four muscles simultaneously, we aim to understand the synergistic muscle activities that characterize knee movement in both healthy individuals and those affected by KOA. The signals were analyzed in parallel, considering the unique contributions of each muscle to knee stability and movement. This parallel analysis allows us to identify patterns that might be indicative of KOA, taking into account the complex interplay between different muscles around the knee. Some specific metrics like accuracy, sensitivity, specificity, and area under the receiver operating characteristic curve can be used to quantify the results.

Furthermore, investigations with a larger patient cohort can compare classification outputs with clinical assessments and imaging modalities, shedding light on the approach’s clinical relevance and practical utility. Long-term follow-up research can be used to evaluate the proposed method’s ability to predict disease progression and treatment response. By gathering user feedback and expert opinions, assessing the proposed system’s effectiveness and interpretability is also possible. These evaluations will shed light on the impact and efficacy of applying deep learning to EMG data to identify KOA.

## Methods

**Fig 1:**
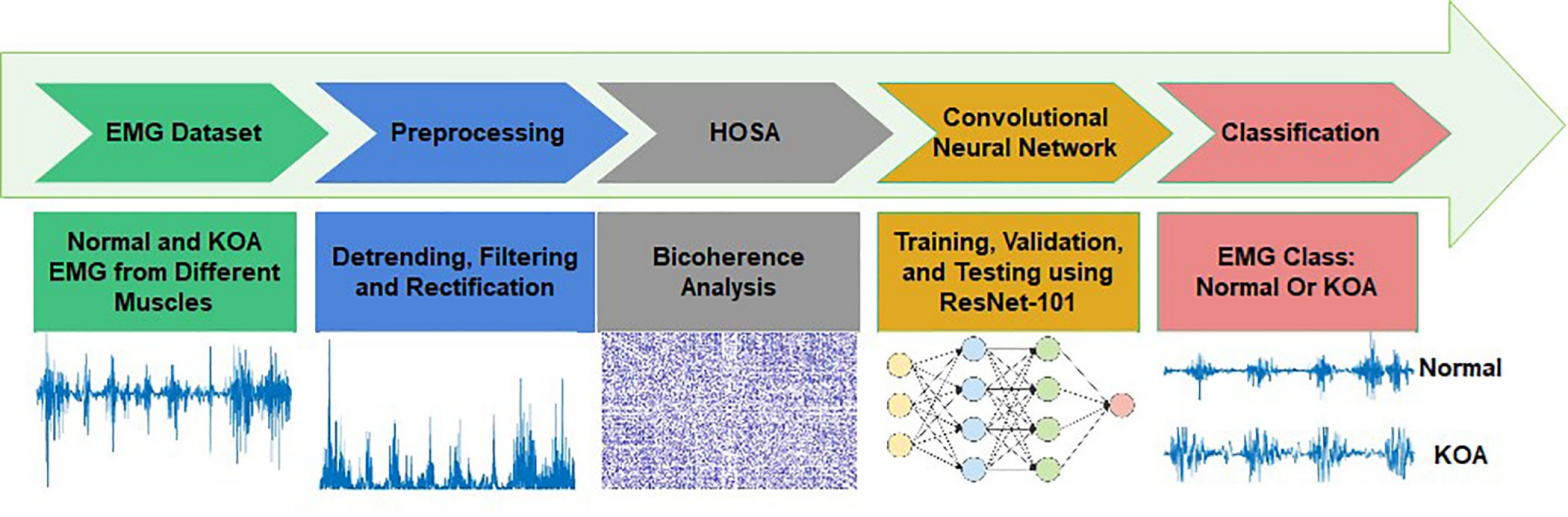
Block diagram of the proposed approach.

This study’s methodology focuses on developing and implementing innovative algorithms for achieving the intended outcomes. The first step of the proposed method is to collect Normal and KOA EMG data from various muscles. Utilize a signal processing technique to process the data, aiming to reveal hidden patterns within the signal. Apply a deep learning network to classify the results that emphasize the differences among the categories to facilitate its classification. Fig 1 depicts the three primary processes of the developed method for EMG type detection: preprocessing, high-order spectrum analysis (HOSA), and diagnosis and recognition using deep learning.

### Dataset description

The EMG signals analyzed in this study were acquired from a publicly available dataset described by Roelker et al [38]. The choice to use a publicly available dataset by Roelker et al. was driven by the dataset’s relevance, quality, and comprehensive documentation of EMG signals under conditions pertinent to the study’s objectives. The dataset encompasses EMG signals collected from 20 subjects during walking at their preferred pace for at least five walking trials. The subjects were ten adults (63.5 ± 3.4 years) without KOA (Normal) and ten adults with KOA (64.0 ± 4.0 years). The healthy subjects did not have any record of any knee injuries and were able to walk without pain or limp. The patients with KOA had suffered from knee KOA, with grade 3 or 4 OA, and were diagnosed by three orthopedic surgeons at The Ohio State Wexner Medical Center. The Telemyo DTS System (Noraxon, Scottsdale, AZ) was employed for signal collection, capturing data from eight key leg muscles at a sampling rate of 1500 Hz. Prior to analysis, signals underwent filtering through a 30–300 Hz sixth-order Butterworth band-pass filter to ensure the removal of frequencies not pertinent to muscle activity, thereby refining the quality of the data for subsequent deep-learning analysis.

**Fig 2:**
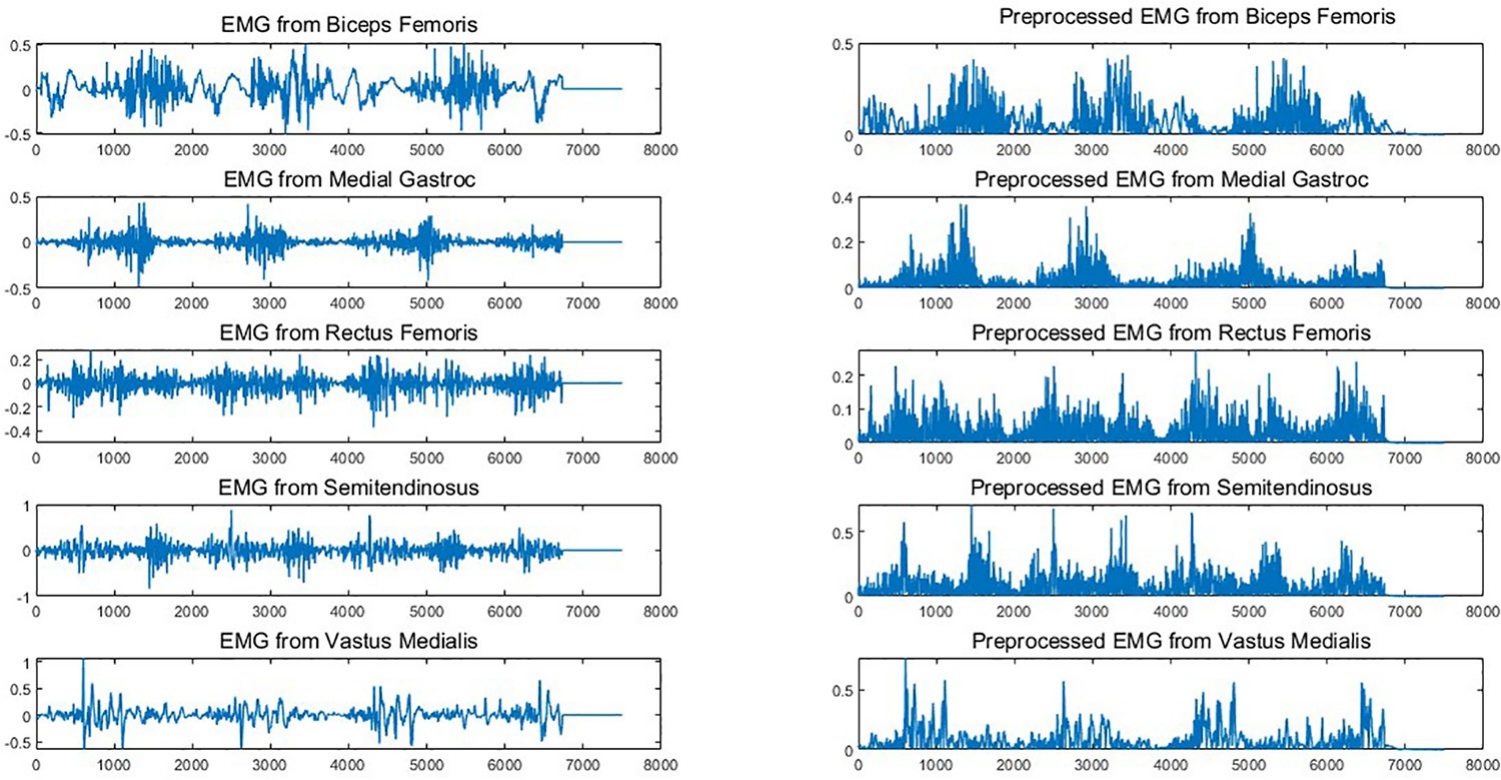
EMG data from five selected muscles before and after preprocessing for the KOA class.

This paper selects Five muscles to build an automated classification system: biceps femoris (BF), medial gastrocnemius (MG), rectus femoris (RF), semitendinosus (ST), vastus medialis (VM), for both EMG types. One sample for each muscle is illustrated in Fig 2.

### EMG preprocessing

Motion artifacts are a substantial source of interference for the surface EMG signal in almost all biomedical signals. The EMG signal and the motion artifacts often have a high-amplitude peak next to each other, but the motion artifacts typically occur at low frequencies. Therefore, a high pass filter was used to remove the artifacts effects [39]. The signal was filtered with a Notch Filter of 60 Hz to remove the DC power line interference, a common artifact observed in EMG signals due to electrical noise from the power supply [22]. This preprocessing step is crucial for ensuring the accuracy of EMG signal analysis by eliminating extraneous noise that can obscure the true physiological signals of interest. This approach is consistent with established practices in EMG signal processing, where mitigating power line interference is recognized as essential for reliable data analysis. Then, EMG signal is rectified to determine the strength of the neural drive to the muscle, which is related to the force of the muscular contraction and its output [40]. All EMG processing was computed on MATLAB® R2022b.

### High Order Spectral Analysis (HOSA)

**Fig 3:**
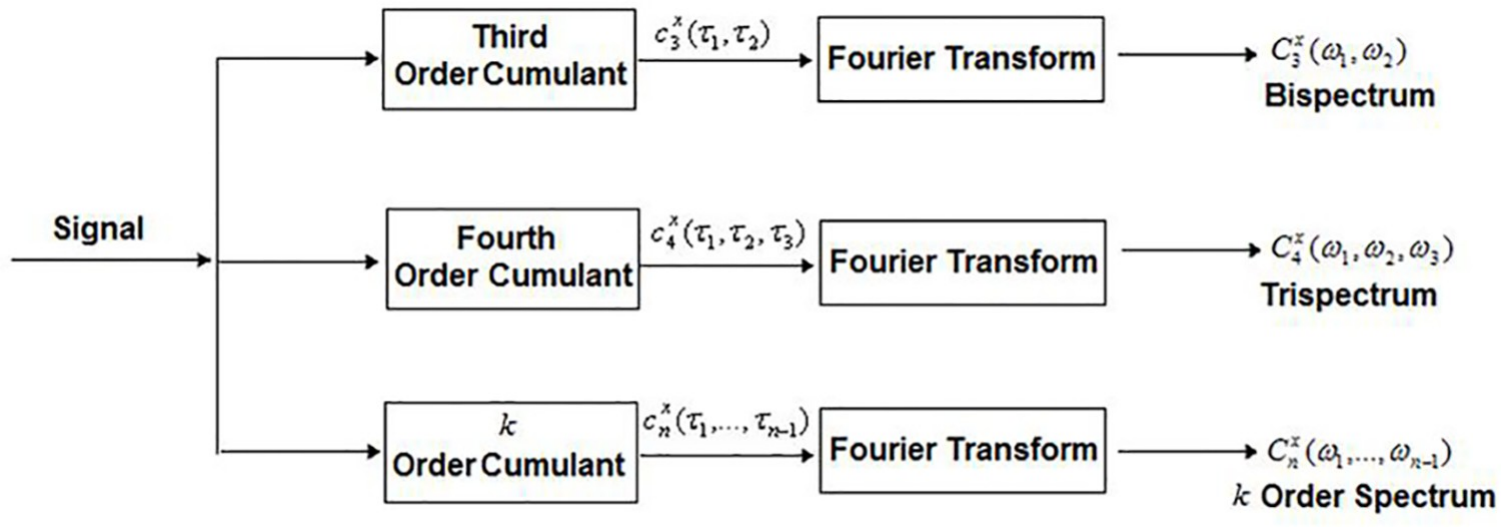
The various higher-order spectra for a deterministic signal. F [.] denotes the k-dimensional Fourier Transform.

HOSA was used for feature extraction and simplifying the complex nature of EMG signals. Higher-order statistics ("cumulants") of a signal are used to generate higher-order spectra (sometimes called polyspectra). Higher-order spectra include the trispectrum (fourth-order spectrum), defined as the Fourier transform of the fourth-order statistics of a stationary signal, and the bispectrum (third-order spectrum), defined as the Fourier transform of the third-order statistics. Fig 3 shows a discrete-time signal’s higher-order spectrum classification map [41, 42]. In contrast to the power spectrum, higher-order spectra are functions of two or more independent frequencies. Numerical spectrum estimates at higher orders may or may not be statistically significant, but they will always be greater than zero. The larger the degrees of freedom, the more reliable the estimate. The amplitude of the higher-order spectrum is essential when studying phase coupling between Fourier components, which holds regardless of the powers of the component frequencies. Coupling the phases by comparing the strengths at the individual frequencies is possible. Normalized spectra can be used to detect and describe nonlinearity in systems because nonlinear interactions produce phase-coupled power at the sum and difference frequencies [43]. The power spectrum (n = 2) and the cumulate spectrum (order n) are combined to create the normalized higher-order spectrum or nth-order coherency (bicoherence) index. The definition of the third-order coherence of a signal with discrete time intervals is:

$$B_{X}(Bicoherence)(f_{1},f_{2})=\frac{B_{x}}{\sqrt{P_{X}(f_{1})P_{X}(f_{2})P_{X}({f_{1}+f}_{2})}}$$ (1)

**Fig 4:**
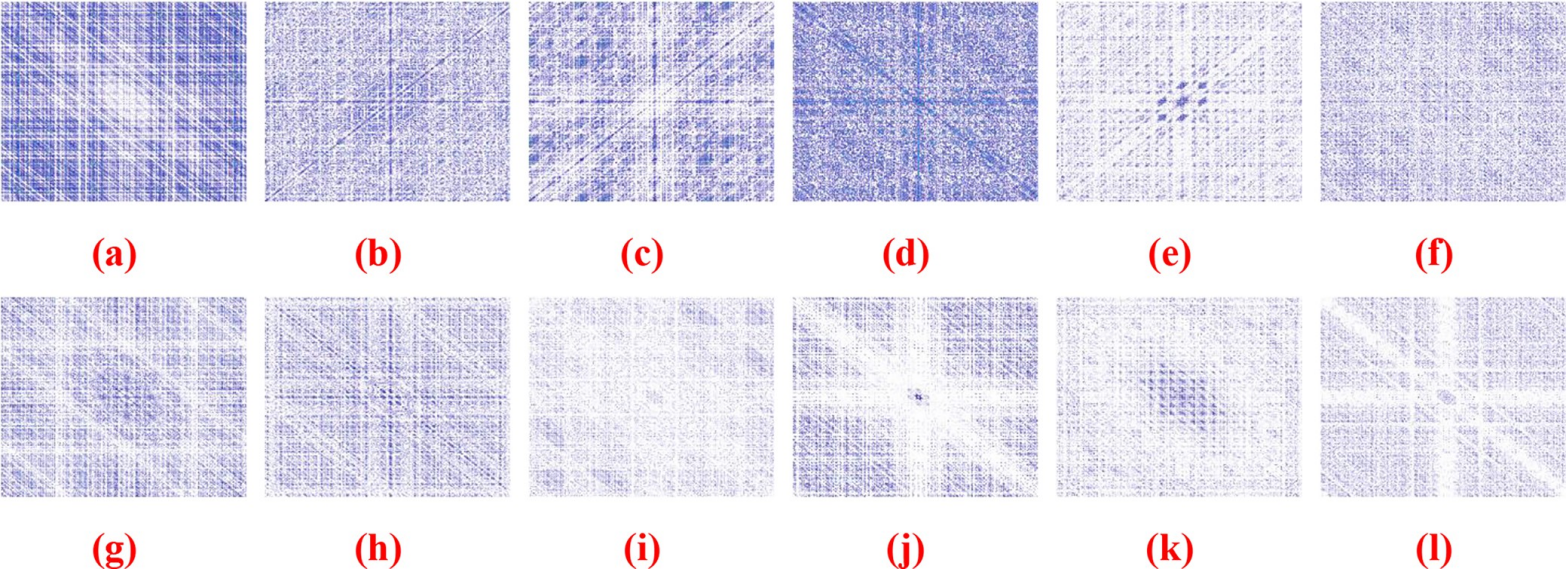
Samples of a Bicoherence representation for EMG signal from five muscles: (a) The Normal EMG from Biceps Femoris; (b) The Normal EMG from Medial Gastroc; (c) The Normal EMG from Rectus Femoris; (d) The Normal EMG from Semitendinosus; (e) The Normal EMG from Vastus Medialis; (f) The Normal EMG from average muscles; (g) The KOA EMG from Biceps Femoris; (h) The KOA EMG from Medial Gastroc; (i) The KOA EMG from Rectus Femoris; (j) The KOA EMG from Semitendinosus (k) The KOA EMG from Vastus Medialis; (l) The KOA EMG from average muscles.

With this function, the power spectrum can be estimated more precisely, and the bispectrum and bicoherence estimates coincide. Auto-bicoherence and cross-bicoherence with the bicoher and bicoherx procedures can be calculated [44]. The HOSA Toolbox in MATLAB® provides the bicoherence function in two classes of electromyography (EMG) from five muscles; the bicoherence representation is formed, and the resulting images are stored with their respective classes, as shown in Fig 4. The deep-learning models will then process the stored images.

### Convolutional neural network

CNN is the best option for image-based classification due to its self-feature learning capabilities and higher classification results on multi-class classification problems. Many researchers have begun using transfer learning approaches to fine-tune the pre-trained deep learning structures for the desired job [45–48].

To find the optimum model for the suggested technique, an adapted version of the pertained Resnet101 CNN model is used in this research. The network has been implemented in MATLAB®. The ResNet101 CNN model was chosen due to its deep architecture of 101 layers, facilitating feature extraction and learning from the complex patterns inherent in the bicoherence images of EMG signals. The transfer learning techniques were utilized to fine-tune the pre-trained ResNet101 model to our specific task. This approach enables the model to leverage knowledge gained from a related task (image recognition on ImageNet) and apply it to our domain-specific problem of classifying EMG signals for KOA diagnosis. The fully connected layer of ResNet101 was adapted to output two classes, corresponding to normal and KOA conditions. This modification ensures that the model’s outputs are directly relevant to our classification problem.

**Fig 5:**
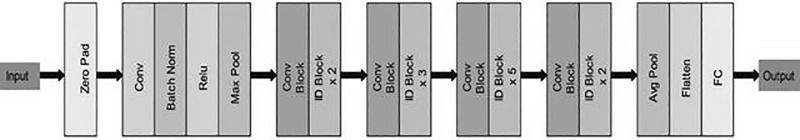
The architecture of ResNet101.

Fig 5 describes the detailed structure of ResNet101 [49]. This preprocessing step ensures that the model can effectively process and learn from our dataset. The data is divided into 70% training, 15% validation, and 15% randomized testing. The study employed a repeated random subsampling validation method, wherein the EMG dataset was randomly partitioned into training and testing sets across five separate iterations. For each iteration, the dataset underwent preprocessing to ensure data cleanliness before being divided. This approach allowed to assess the robustness and consistency of the proposed machine learning classifiers’ predictive performance on different subsets of the data, thereby providing a reliable estimate of their generalization ability to unseen data. The model is built by the following hyperparameters; Adaptive Moment Estimation (Adam) for efficient gradient descent, mini patch size 32, maximum epochs 60, the initial learning rate 0.001, and the validation frequency 3. All images fed into the model were standardized to a size of 224×224×3, aligning with the input dimensions expected by adapted version of the ResNet101.

### Statistical analysis

The results are presented as mean with standard deviations. Statistical analysis was performed using Statistical Package for the Social Science (SPSS, v.21.0, SPSS Inc, Chicago, IL). A one-way analysis of variance (ANOVA) test was used to evaluate whether the type of muscle (e.g., medial gastrocnemius, rectus femoris, etc.) significantly affects the machine learning model’s performance in predicting KOA. The test specifically assesses the effect of muscle type on various performance metrics of machine learning models, such as accuracy, sensitivity, and specificity. Tukey’s Honest Significant Difference (HSD) post-hoc test was used to investigate the main effect of the type of muscles on different machine learning performance metrics. Probability (p) values < 0.05 were considered statistically significant.

## Results

**Fig 6:**
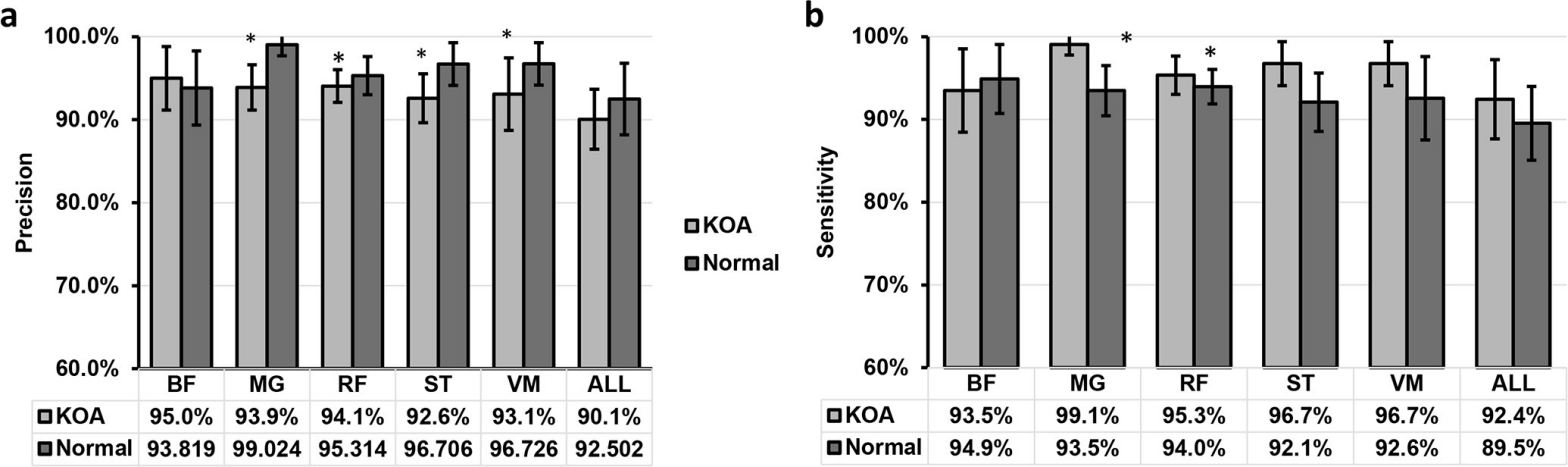
(a) Precision and (b) sensitivity for prediction the knee osteoarthritis (KOA) and normal subjects using EMG signals from different muscles. Where biceps femoris (BF), medial gastrocnemius (MG), rectus femoris (RF), semitendinosus (ST), vastus medialis (VM), and average of all five muscles (ALL) groups. Data are presented as mean with standard deviations (mean ±STD). *P<0.5, significantly higher than ALL group.

In this study, a total of 20 subjects consisting of normal subjects and subjects with KOA in equal numbers were examined. The dataset was divided into 70/15/15 Train-Validate-Test datasets. The datasets are subsequently trained on a variety of machine-learning classifiers. The data were trained on machine learning classifiers at different five runs. In each run, the datasets were selected arbitrarily and put in train, validation or train set. For each muscle, the confusion matrices were obtained for each run and the features were extracted and further analyzed. In addition to that, the EMG signals from the five muscles together were used to predict the KOA. Fig 6 shows the precision and sensitivity results for the prediction of normal and KOA subjects using machine learning based on EMG signals from different muscles. The results showed that the average precision where more than 90% when using the EMG signals from any of the five muscles and all muscles for both normal and KOA subjects. Medial gastrocnemius (MG), rectus femoris (RF), semitendinosus (ST), and vastus medialis (VM) have significantly higher precision than using all the muscles to predict KOA. Moreover, the sensitivity was more than 92% for prediction KOA for all the muscles, in which the MG muscle has 99.1% followed by ST and VM with 96.7% average sensitivity. Using all muscles together to predict the normal subject has a lower sensitivity of 89.5%. MG and RF had significantly higher sensitivity than using all muscles to predict the normal person (Fig 6B).

**Fig 7:**
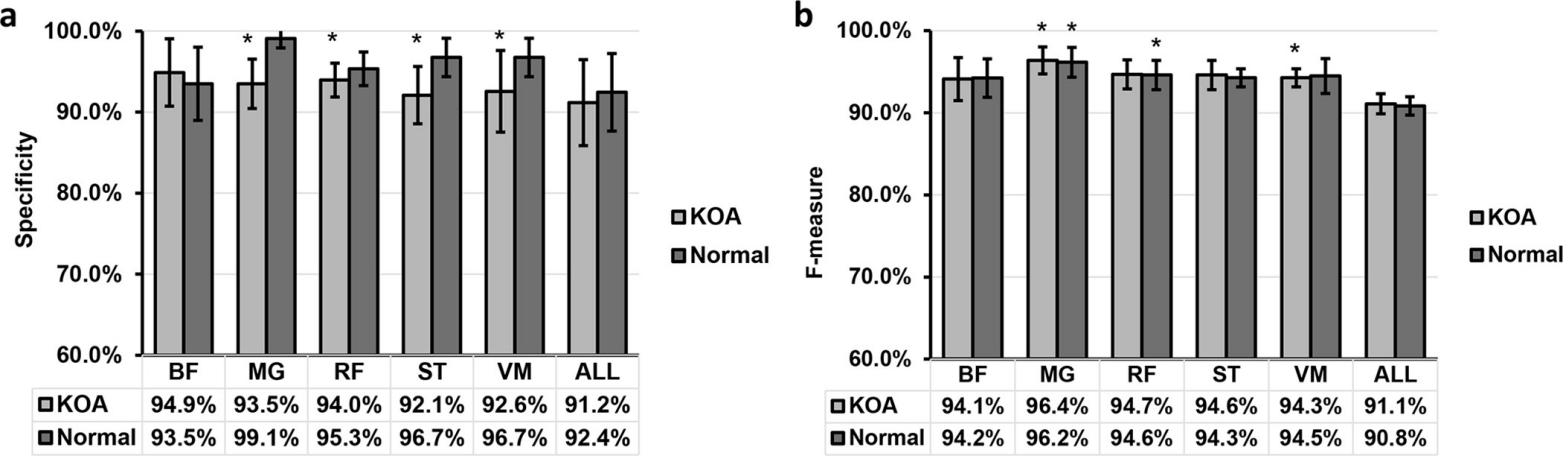
(a) Specificity and (b) F-measure for prediction the knee osteoarthritis (KOA) and normal subjects using EMG signals from different muscles. Data are presented as mean ±STD. *P<0.5, significantly higher than ALL group.

The specificity and the F-measure for predicting knee abnormality from EMG signals are shown in Fig 7. The average specificity is more than 93% for BF, MG, and RF for prediction KOA. While the average specificity is more than 96% for ST and VM for the prediction of normal knee. Moreover, the specificity for using MG, RF, ST and VM are significantly higher than using all the muscles together to predict KOA. The F- measure is the highest for MG for both prediction KOA and normal subjects with 96.4% and 96.2% respectively. Moreover, the F-measure is significantly higher for MG muscles as compared with all muscles for both prediction KOA and normal subjects. VM muscle has a significantly higher F-value as compared with all muscles for prediction KOA.

Table 1 shows the Area Under the Curve (AUC), Matthews correlation coefficient (MCC) and accuracy for all the fusion matrices to predict knee osteoarthritis (KOA) and normal subjects using EMG signals. AUC values are more than 0.98 for all muscles when are used individually to predict the subjects with KOA. While the AUC is 0.964 when the average of all muscles is used for prediction. AUC values are significantly higher for MG, ST and VM as compared to ALL. MCC values are significantly higher for MG and VM as compared to ALL group. The average accuracy is 94.2%, 96.3%, 94.7%, 94.4%, 94.7% and 91% for BF, MG, RF, ST, VM, and ALL muscles respectively. Interestingly, MG has the highest accuracy between the average for all the muscles and has significantly higher accuracy than the average of all the muscles together for prediction abnormality. The one-way ANOVA test’s findings, as applied in this study, suggest that certain muscles, such as the medial gastrocnemius, may have a more pronounced role in accurately predicting KOA based on EMG signals. This is evidenced by higher precision, sensitivity, and specificity values compared to other muscles or the aggregate of all studied muscles.

**Table 1:** Area Under the Curve (AUC), Matthews correlation coefficient (MCC) and accuracy using different muscles to predict knee osteoarthritis (KOA) and normal subjects using EMG signals.

Muscles	BF	MG	RF	ST	VM	ALL
**AUC**	0.985±0.018	0.994±0.008*	0.984±0.015	0.994±0.004*	0.993±0.004*	0.964±0.019
**MCC**	0.886±0.048	0.927±0.034*	0.893±0.035	0.891±0.018	0.896±0.037*	0.823±0.024
**Accuracy(%)**	94.2%±2.5	96.3±1.7*	94.7±1.8	94.4±1	94.7±1.9	91±1.1

*P<0.5, significantly higher than ALL group.

## Discussion

In this study, EMG signals from five different muscles were used to discriminate KOA subjects from normal controls using a machine learning technique, which could give a quantitative tool for KOA diagnosis. Our results indicate that the EMG signal from the medial gastrocnemius (MG) muscle was able to distinguish KOA patients from normal with 96.3% accuracy and 0.994 AUC. Also, the EMG signals from the biceps femoris (BF), rectus femoris (RF), semitendinosus (ST) and vastus medialis (VM) were able to distinguish KOA patients from normal with around 94% accuracy while when the five muscles together yielded only 91% accuracy. While it is acknowledged that EMG signals inherently possess noise due to motion artifacts, baseline fluctuations, and interference [18]. It is essential to highlight the efficacy of advanced signal processing and deep learning techniques employed in this study to mitigate these challenges. These methods significantly enhance the signal-to-noise ratio, allowing for the extraction of meaningful information from the EMG data that is relevant to diagnosing KOA. Furthermore, MRI, despite being the gold standard for visualizing joint structures and assessing OA severity, does not provide direct insights into the dynamic muscle activities and neuromuscular control associated with knee function and pathology [50]. EMG fills this gap by offering real-time, non-invasive measurements of muscle activity, which are crucial for understanding the biomechanical and functional aspects of knee OA. The complementary nature of EMG and MRI highlights the multidimensional approach required for a comprehensive assessment of KOA. While MRI excellently depicts structural changes, EMG provides a unique window into functional impairments, allowing for a holistic understanding of the disease’s impact on knee joint stability and movement.

Machine learning and deep learning techniques were used to predict the KOA in different studies. Many studies have used the same datasets from UCI machine learning repository by Sanchez et al [51] to predict knee abnormality. EMG signals from RF, BF, VM and ST muscles from 11 healthy subjects and 11 subjects with knee abnormalities were collected during standing, walking, and sitting. In Vijayvargiya et al., Five different classifiers were used in the study including k-nearest neighbor, support vector machine, decision tree, random forest and extra tree [33]. Forty-four features were extracted from four different muscle EMG signals then the backward elimination technique was used to select the relevant features by the p-value test. The highest accuracy was 91% in detecting knee abnormality and achieved by Extra Tree Classifier. In another study, the EMG signals were denoised by the Wavelet Denoising method followed by the extraction of eleven features, then using oversampling to balance the data [52]. After that different classifiers were used such as the extra tree, SVM, MLP, random forest and gradient boosting with extra tree have the highest accuracy (92.5%). In another study with the same dataset, anomaly detection techniques were used to improve the performance of the classifiers [32]. The accuracy was 98.5% when using the iforest anomaly detection method on the light gradient boosting machine model while the accuracy was as low as 85% without any adjustment methods. Zhao et al. [53] have used XGBoost and cross-validation (CV) to predict the KOA from EMG signals and compare them to SVM and the deep neural network (DNN). XGBoost classifier has higher accuracy than the other two methods (the average accuracy of ten experiments was 96.09%). In our study, the accuracy of prediction the knee OA was higher than 94% when using EMG signals from BF, RF, VM, ST or MG muscles. Previous studies, such as those by Vijayvargiya et al. [33, 52], have indeed explored the potential of machine learning and deep learning techniques for differentiating healthy subjects from those with KOA using EMG signals. Notably, studies have often analyzed EMG signals in isolation or employed conventional machine learning techniques that may not fully capture the complex, non-linear interactions between muscle activities during knee movement. This study advances this research by not only employing a deep learning approach but also by integrating it with the bicoherence analysis of EMG signals. This integration allows for a more advanced understanding of the non-linear interactions between muscle activities, which is critical for accurately diagnosing KOA. The bicoherence method provides a novel way to feature EMG signals for deep learning analysis, enhancing the model’s diagnostic capability beyond what has been reported in previous studies.

Chen et al. have used EMG signals from VM, ST, BF, and vastus lateralis (VL) muscles to distinguish between KOA subjects and control based on three entropy measures, i.e., approximate entropy, sample entropy, and fuzzy entropy [54]. They found that using fuzzy entropy features from the EMG signals of VM and BF muscles yielded 92% accuracy, 91.43% sensitivity and 93.33% specificity in distinguishing KOA subjects. Parisi et al. have used a Genetic Algorithm-based denoising approach on the EMG signals to maximize mutual information and minimize entropy [55]. The EMG signals of RF, BF, VM, ST, MG, hamstrings medial (MH) and lateral gastrocnemius (LG) muscles were encompassed in the study. The use of Genetic Algorithm-Denoising- Lagrangian Support Vector Machine (GA-D-LSVM) resulted in very high accuracy (99.57%). It should be pointed out that EMG signals from 7 different muscles were used to train the model and the data used in the study were selected from two databases. In this article, the pre-processing steps were conducted to enhance the quality of the signal and did not eliminate frequency-related features or outliers data or use anomaly detection. The obtained results of this study demonstrated that the MG muscle exhibits distinct patterns that are highly predictive of KOA, achieving 96.3% accuracy, while BF, RF, ST and VM muscles were able to predict KOA with around 94% accuracy. A study by Derek et al. has shown that subjects with moderate KOA had higher and prolonged quadriceps and higher lateral hamstring activity as compared to a control subject [56]. Interestingly, they found that the activity of both lateral and medial gastrocnemius (MG and LG) muscles increased during the early stance phase which could enhance joint stability during weight acceptance and single-leg stance, especially in severe KOA. This could explain the obtained results that show MG has the highest prediction accuracy of KOA. In a meta-analysis review, they found that patients with KOA have augmented co-contraction, amplitude and duration of lateral knee muscles [57]. RF, VL and BF activation amplitudes were usually increased in moderate KOA patients.

Table 2 provides comparative performance results of the proposed method with recently published articles. This comparison demonstrates the advanced accuracy, sensitivity, specificity, and AUC metrics achieved by this study, particularly highlighting the utility of combining high-order spectral analysis (HOSA) with deep learning adapted (ResNet101) for diagnosing knee osteoarthritis (KOA) through EMG signals. Furthermore, this juxtaposition against clinical evaluations and other EMG methods serves as external validation, reinforcing the reliability and applicability of our findings. By showcasing how the method aligns with or surpasses the diagnostic capabilities of existing approaches, these findings underscore its potential contribution to the field. Notably, the superior performance metrics—especially the prediction accuracy of the medial gastrocnemius (MG) muscle—underscore the method’s diagnostic precision. Conversely, the study represents a significant step forward in this endeavor, offering new insights into the neuromuscular alterations associated with KOA and paving the way for future research to explore these methods in clinical settings

**Table 2:** Comparison of the proposed method’s performance with other studies.

Study	Methods	Muscles	Accuracy
Chen et al. [54]	fuzzy entropy	VM and BF	92%
Vijayvargiya et al. [33]	Extra Tree Classifier	RF, BF, VM and ST	91%
Vijayvargiya et al. [52]	Extra Tree Classifier	RF, BF, VM and ST	92.5%
Parisi et al. [55]	GA-D-LSVM	RF, BF, VM, ST, MG, MH and LG	99.57%
Zhao et al. [53]	XGBoost	RF, BF, VM and ST	96.09%
This study	HOSA- ResNet101	MG	96.3%

Notably, the classification problem for the four grades of KOA must be addressed, and the deep learning model must be strengthened to withstand the difficulty of distinguishing between healthy knees and lower-grade osteoarthritis. Future research could therefore be directed in accordance with the subsequent strategies: Expand the sample size to encompass a more extensive spectrum of knee osteoarthritis (KOA) grades, ranging from Grade 1 (severe) to Grade 4 (doubtful). Convert the deep learning model to handle a multi-class classification problem, requiring it to categorize knees into the following five groups: normal, Grade 1, Grade 2, Grade 3, and Grade 4 KOA. Investigate sophisticated feature engineering methodologies in order to extract more informative features from EMG signals that are capable of distinguishing between various grades of KOA. A number of critical characteristics, including fatigue resistance, co-contraction, and muscle activation patterns, may differ between categories and are therefore essential for precise classification. To mitigate the lack of diversity in the training dataset, employ data augmentation methods that are tailored to EMG signals, including amplitude scaling and time distortion. It is advisable to integrate deep learning with conventional machine learning methods, which have demonstrated potential in comprehending the evolution of KOA. A hybrid approach has the potential to capitalize on the respective merits of both methodologies in order to enhance the precision and dependability of diagnostics. Incorporate clinical insights and additional non-EMG data into the model, such as patient demographics, duration of symptoms, and physical examination findings. The utilization of this multimodal approach may facilitate the differentiation of KOA grades by offering a more comprehensive view of the disease. Conduct external validation on the model by employing distinct datasets that were not utilized during the phases of training or testing. Incorporate the knowledge and skills of radiologists, clinicians, and physiotherapists through collaborative efforts during the model development and validation phases. With their valuable insights, the model’s output can be enhanced to better suit clinical needs and requirements. Through the implementation of these strategies, a more resilient and dependable deep-learning model can be constructed to categorize KOA grades. Such a model would make a substantial contribution to the timely detection, surveillance, and individualized treatment of patients afflicted with KOA.

## Conclusion

In conclusion, higher-order spectral analysis (HOSA) techniques were able to enhance feature extraction of the EMG signals from five knee muscles. Bicoherence representation of EMG signals was fed to a modified deep learning network. The combination of bicoherence and adabted ResNet 101 enabled the achievement of robust metrics for accuracy, precision, and sensitivity. The 5 muscles were able to distinguish Knee OA patients from normal with very good accuracy. Interestingly, the medial gastrocnemius (MG) muscle has the highest prediction accuracy. A future research direction could involve expanding the dataset to include a larger number of subjects from diverse demographics, including varying ages, genders, and stages of KOA. This expansion would allow for a more comprehensive analysis of the deep learning model’s performance across a broader spectrum of the population and enhance the model’s generalizability. Additionally, analyzing the dataset for gender-specific patterns in EMG signals related to KOA could provide insights into personalized treatment strategies, potentially leading to more targeted and effective interventions for different patient groups.
